# Sex Difference in Oxytocin-Induced Anti-Hyperalgesia at the Spinal Level in Rats with Intraplantar Carrageenan-Induced Inflammation

**DOI:** 10.1371/journal.pone.0162218

**Published:** 2016-09-08

**Authors:** Lok-Hi Chow, Yuan-Hao Chen, Wan-Chuan Wu, En-Pei Chang, Eagle Yi-Kung Huang

**Affiliations:** 1 Department of Pharmacology, National Defense Medical Center, Nei-Hu, Taipei, Taiwan, ROC; 2 Department of Anesthesiology, National Defense Medical Center, Taipei, Nei-Hu, Taiwan, ROC; 3 Department of Anesthesiology, Taipei Veterans General Hospital, Taipei, Taiwan, ROC; 4 Department of Anesthesiology, National Yang-Ming University, School of Medicine, Taipei, Taiwan, ROC; 5 Department of Neurological Surgery, Tri-Service General Hospital, National Defense Medical Center, Taipei, Nei-Hu, Taiwan, ROC; University of California Los Angeles, UNITED STATES

## Abstract

Previously, we demonstrated intrathecal administration of oxytocin strongly induced anti-hyperalgesia in male rats. By using an oxytocin-receptor antagonist (atosiban), the descending oxytocinergic pathway was found to regulate inflammatory hyperalgesia in our previous study using male rats. The activity of this neural pathway is elevated during hyperalgesia, but whether this effect differs in a sex-dependent manner remains unknown. We conducted plantar tests on adult male and female virgin rats in which paw inflammation was induced using carrageenan. Exogenous (i.t.) application of oxytocin exerted no anti-hyperalgesic effect in female rats, except at an extremely high dose. Female rats exhibited similar extent of hyperalgesia to male rats did when the animals received the same dose of carrageenan. When atosiban was administered alone, the severity of hyperalgesia was not increased in female rats. Moreover, insulin-regulated aminopeptidase (IRAP) was expressed at higher levels in the spinal cords of female rats compared with those of male rats. Oxytocin-induced anti-hyperalgesia exhibits a sex-dependent difference in rats. This difference can partially result from the higher expression of IRAP in the spinal cords of female rats, because IRAP functions as an enzyme that degrades oxytocin. Our study confirms the existence of a sex difference in oxytocin-induced anti-hyperalgesia at the spinal level in rats.

## Introduction

Oxytocin (OT) has been characterized as a key hormone in the body [1]. The idea of using OT to treat pain is not novel; in numerous clinical and animal studies, systemic and central OT treatments have shown that OT can act as an effective analgesic [2–7]. In 1987, Madrazo et al. first demonstrated that intracerebroventricular administration of OT can produce a substantial analgesic effect in terminal cancer patients [8]. In 1994, Yang reported that intrathecal (i.t.) injection of OT was effective in providing pain relief to patients with chronic low back pain [9], this analgesic effect of OT lasted up to 5 hours. The study also showed that i.t. administration of OT exerted a similar antinociceptive effect in rats, which could be blocked using an OT-receptor (OTR) antagonist and naloxone [9]. However, Takagi et al. reported that the levels of OT in the cerebrospinal fluid and plasma were not correlated in humans [10], which suggested the existence of independent peripheral and central pools of OT. Thus, OT might not be able to cross the blood-brain barrier under normal conditions.

During the past decade, an OT neural pathway originating from the paraventricular nucleus (PVN) and projecting to the spinal cord was identified and determined to be a new anti-nociceptive pathway [11]. In 2001, a descending OT pathway was first revealed in female rats, although previous studies had indicated a potential analgesic effect of OT at the spinal level [9,12,13]. OT, which is possibly released under the control of endogenous opioids, also mediates stress-induced analgesia in the spinal cords of adult mice [14,15]. In 2003, Yu et al. reported the involvement of OT in spinal antinociception in rats with intraplantar carrageenan-induced inflammation [16]. This antinociceptive effect of OT could be attenuated using mu- and kappa-opioid antagonists. These findings indicated the importance of the PVN-spinal cord OT neural pathway in the regulation of pain in rats with hyperalgesia. Stimulation of the PVN suggested that this OT neural pathway is a descending pathway that can be activated under various pathological pain-producing conditions [17–19]. Electrophysiological studies have shown that OT plays a role in neuropathic pain [20]: OT produced a clear anti-hyperalgesic effect in neuropathic rats with spinal nerve ligation (SNL), which could be caused by the presence of sensitized OTRs in spinal dorsal horns [20]. Collectively, these studies indicated that the PVN-spinal cord OT neural pathway could be a critical regulator of the development of inflammatory hyperalgesia and neuropathic allodynia.

Although peripheral OT has also been reported to function in anti-inflammation [21], in a previous study, we focused on the effect of OT at the spinal level [22]. We demonstrated that i.t. administration of OT produced a marked anti-hyperalgesic effect in male rats with carrageenan-induced inflammatory hyperalgesia [22]. However, the oxytocinergic system has been demonstrated to exhibit sexual dimorphism in the brain, which may cause differences in behavioral expression (e.g., social behavior) [23]. However, whether sex differences also exist at the spinal level in this newly identified pain-regulating OT pathway remains unclear. Therefore, we investigated the effects of OT in the same carrageenan-induced hyperalgesia model by using female rats, and then compared these data with those obtained from age-matched male rats. This enabled us to examine the sex-dependent differences in OT-induced anti-hyperalgesia at the spinal level. We also investigated the potential effect of PVN stimulation on hyperalgesia in female rats. The PVN-stimulation model is well established and widely used [22]. Thus, the results of our experiments could verify the possible function of the descending OT pathway in anti-hyperalgesia in female rats.

To identify the mechanisms that might underlie a sex difference in the OT pathway, we semi-quantified the levels of insulin-regulated aminopeptidase (IRAP) in the spinal cords of male and female rats. IRAP is recognized to function as a cell membrane-bound enzyme, and peptide ligands (angiotensin IV and LVV-hemorphin 7) can block IRAP activity [24]. Although IRAP can act on numerous substrates, it also functions as a key endogenous enzyme that degrades OT [25,26]. Thus, potential disparities in IRAP expression could cause a sex difference in the OT pathway, although other factors such as levels of OT-R expression could also be critical [27].

## Methods

### Animals

Pregnant Sprague-Dawley (S.D.) rats were purchased from BioLASCO Taiwan Co., Ltd. The pregnant rats purchased were all 2 weeks from mating. After birth which is roughly 1 week following accommodation, pups were kept with the dam until Post-natal Day 21 (P21). Male and female rats were separated and kept 3 per cage. In this study, 8-wk-old virgin female and male rats were used. All animals were bred in the Animal Facility of the National Defense Medical Center. The animal rooms were maintained at 23 ± 2°C in a 12-h light/dark cycle, and food and water were available to the animals ad libitum throughout the experiment. Pellets of standard diet (Laboratory Rodent Diet 5001, LabDiet, MO, U.S.A.) was used to feed the animals. The health condition of the animals was monitored daily by professional staffs in the animal house. No observable signs of weight loss, inappetence, weakness/inability to obtain feed or water, moribund state, and infection were found in all the animals that we used throughout the experiments, although we have set the criteria for the early euthanasia according to these signs. None of the animals became ill or died prior to the endpoint of our experiments. Animals were transferred to the testing room in the morning before use in the experiment, which was conducted during the light cycle. The experimental protocol was approved by the Animal Care and Use Committee of the National Defense Medical Center, Taipei, Taiwan, ROC. The reference numbers are IACUC-06-156 and IACUC-09-192.

### Implantation of an intrathecal catheter

The i.t. catheter was implanted using surgical procedures described in our previous reports [22,28,29]. These methods were adopted and modified from the method originally reported by Yaksh and Rudy [30]. Before surgery, rats were anesthetized with pentobarbital (50 mg/kg, i.p.). An i.t. catheter was implanted at the lumbar level for drug administration as described [22,28,29]. Animal were allowed to recover from the surgery for 4 days, and no animal was used in more than one experiment. Any animal that exhibited motor impairment was not used in the study. One day before the experiments, animals bearing the i.t. catheters were injected with 20 μl of 2% lidocaine by using a microsyringe (Hamilton, 25 μl) to induce temporary (10–20 min) motor blockade of the lower limbs to determine whether the catheter was positioned accurately.

### Plantar test in carrageenan-induced inflammation

To induce acute inflammation, 100 μl of carrageenan Type IV (Sigma, USA) solution (1.5%, w/v in saline) was injected into the subcutaneous space of the right hind paw of the rats. Immediately following the injection of carrageenan, the test compounds were injected i.t. into the rats in distinct groups. An Ugo Basile 7371 plantar tester (Italy) was used to measure the withdrawal latency of the paw that received the carrageenan injection. The IR intensity was set at 45 and the cut-off time was 20 s. Basal latency was measured before the intraplantar injection of carrageenan (-1 h), and then paw-withdrawal latencies were measured after the injection at 0, 1, 2, 3, 4, 4.5, 5, 5.5, 6, 7, 24, and 31 h.

### Electrical stimulation at the paraventricular nucleus

The stimulating electrodes and procedures used were similar to those reported by Yirmiya et al. [31] and Miranda-Cardenas et al. [19]. The stimulating electrode was positioned at the parvocellular division of the PVN to ensure the activation of the PVN-spinal cord oxytocinergic pathway. The guide cannula was made of a G19 needle that was cut to remove the sharp end. The cannula was implanted and positioned in the parvocellular division of the PVN at the following coordinates: A -1.40 mm, L 0.2 mm, and V -6.6 mm from the bregma [32]. The cannula was surgically implanted after the rats were anaesthetized with pentobarbital (50 mg/kg, i.p.). The rats were allowed to recover from the surgery for 4 days, and then used in the PVN-stimulation experiments. The stimulating electrodes were purchased from WPI Inc. (TM53CCINS Concentric Bipolar Microelectrode, 127 mm, WPI Inc., USA). The electrodes were fixed in a plastic syringe and allowed to extend only 1 mm out of the guide cannula when inserted for stimulation. This helped ensure that the electrode reached the PVN, because the coordinates of the guide cannula were chosen such that they reached 1 mm above the PVN. The purchased electrodes were made of tungsten and were 127 μm in diameter. The electrodes were coated with Parylene C on their outer surface, and the finest tip end (3–4 μm) was exposed to a length of 0.4 mm in a triangle shape (impedance 10–15 K). Stainless steel tubing was inlaid within the electrodes. These concentric electrodes allowed the delivery of bipolar stimulation in vivo. The electrical PVN stimulation consisted of a train of 1 ms pulses at 60 Hz over a period of 6 s [26]; the pulse train was generated by a Grass stimulator (S88 Dual Output Square Pulse Stimulator, Grass Tech., USA) and delivered through a stimulus isolator (A360LA Stimulus Isolators, WPI Inc.). The intensity of all stimuli was set at 100 μA. Following PVN stimulation, animals were subjected to the plantar test, which was conducted once every 2 min for 10 min and then at 30 min after PVN stimulation. The position of stimulating electrode was examined using postmortem sectioning of the brain to compare it with the coordinates shown in a brain atlas [32].

### Paw edema test

The rats that received intraplantar carrageenan and i.t. injections were subjected to paw-edema tests [33]. Using a water-resistant marker, a blue line was drawn at the ankle joint of the rats. An Ugo Basile 7150 plethysmometer (Italy) was used to measure the volume of the paw that received the carrageenan injection. When measuring the volume of the paw, the blue line was aligned with the surface of the solution (0.5 g NaCl/L H_2_O) in the measuring water-cell to ensure standardization. The paw volume was first measured before the intraplantar injection of carrageenan (-1 h), and the volumes were again measured after injection at 0, 1, 2, 3, 4, 4.5, 5, 5.5, 6, 7, 24, and 31 h.

### Western blotting analysis

Rats were sacrificed by means of decapitation and lumbar spinal-cord tissues (L1 to L6) were carefully dissected and then homogenized in ice-cold lysis buffer (#9803, Cell Signaling Technology, USA) by using an ultrasonic cell-disruptor unit (MICROSON XL2000, Misonix Inc., Farmingdale, NY, USA). For use in experiments on dorsal and ventral spinal cords, the spinal cords were cut horizontally at the middle to separate the dorsal and ventral parts. Total protein with a mixture of cytoplasmic and membrane fractions was isolated using the following steps. The homogenates were centrifuged at 1000 x *g* at 4°C for 10 min, and the supernatants obtained were centrifuged again at 20000 x *g* at 4°C for 60 min. The pellets were re-suspended in the same buffer and the protein concentrations were determined, after which the samples were boiled and 40 μg of total protein was loaded into a lane of 8% polyacrylamide gels. Following electrophoresis, protein in the SDS-PAGE gels was transferred to polyvinylidene fluoride membranes. The membranes were immunoblotted using a polyclonal rabbit antibody against IRAP (1:1000, IRAP11-A; Alpha Diagnostic, USA), and the immunoreactive bands were visualized by incubating the membranes with horseradish peroxidase-conjugated goat anti-rabbit IgGs (1:5000, Cell Signaling Technology) and then using an enhanced chemiluminescence kit (ECL-Plus, GE Healthcare, UK). AccuRuler RGB Prestained Protein Ladder (Cat No.02101-250, Maestrogen, Inc., NV, U.S.A.) was used as the molecular weight markers to check the identity of IRAP. The staining of β-actin was used as an internal control for quantifying protein-expression levels. Using the software ImageJ 1.48v (U.S.A.), the optical densities of stained bands were measured and the IRAP levels were compared after calculating the ratios of the band intensities relative to the corresponding internal standard.

### Statistical analysis

All data are presented as means ± SEM. To examine the significance of differences between groups, we used two-way ANOVA followed by the Bonferroni post-test. When comparing only two groups of data, the paired Student’s *t*-test was used. *P < 0.05 was considered statistically significant.

## Results

### Sex difference in carrageenan-induced hyperalgesia

**Fig 1:**
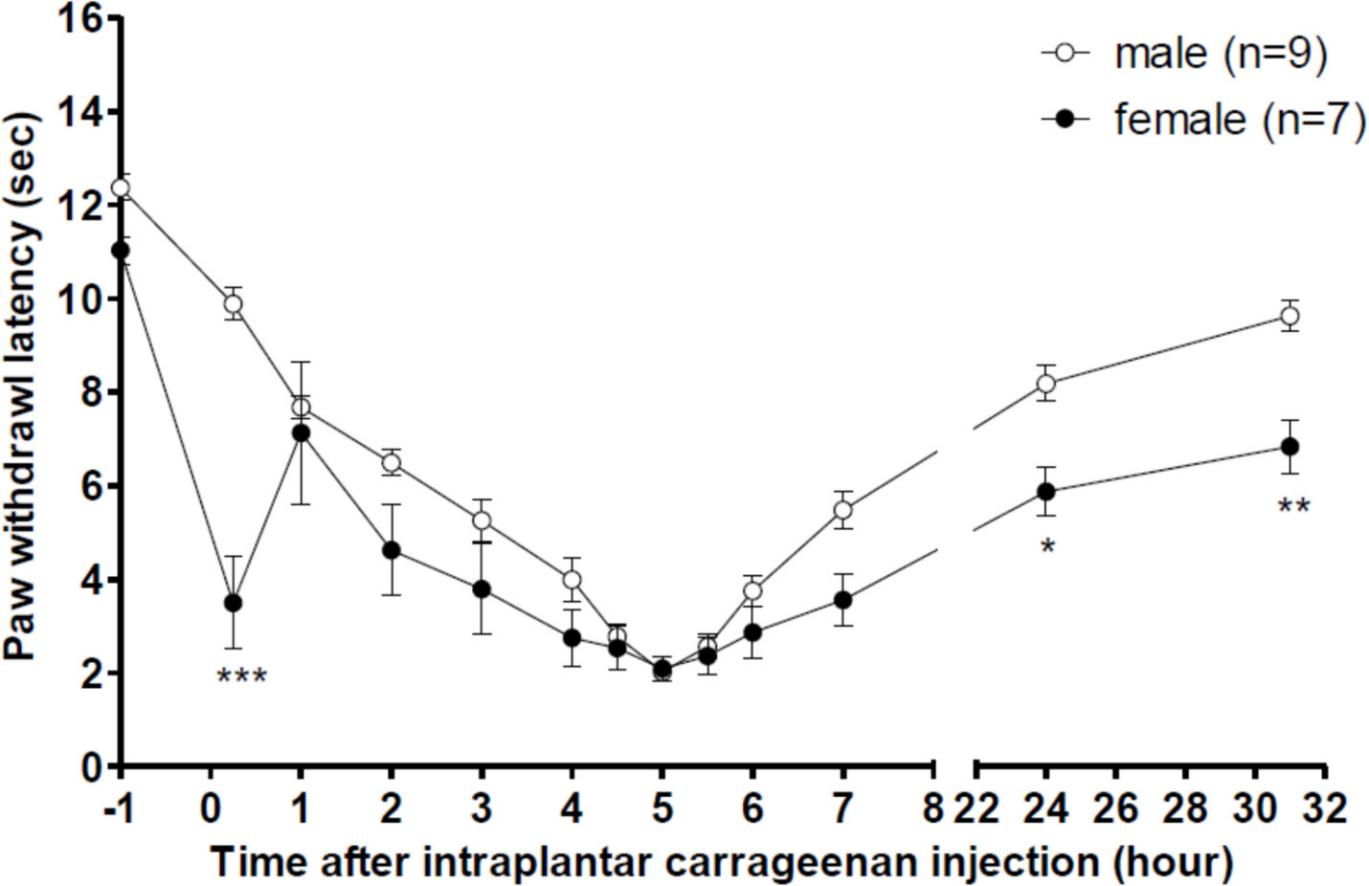
Comparison of thermal hyperalgesia induced by intraplantar carrageenan injections in male and female rats. Data from male and female rats received i.t. injections of saline were placed together for comparison. Two-way ANOVA followed by Bonferroni post-test was used to analyze the statistical significance (*P < 0.05, **p < 0.01, ***p < 0.001, female *vs*. male group).

Intraplantar carrageenan injection caused a significant hyperalgesia in both male and female rats. However, female rats exhibited more severe hyperalgesia than did male rats when the animals received the same carrageenan injection (1.5%), as shown in Fig 1. In this figure, the data obtained from the saline groups of both male and female rats are presented and compared. Following carrageenan injection, the paw-withdrawal latency at 15 min decreased to 3.5 ± 1.0 s in female rats, but the latency only decreased to 9.9 ± 0.4 s in male rats, and this difference was significantly different. This could have occurred because the response to the acute pain caused by the intraplantar injection was more sensitive and unstable in female rats than in male rats. Furthermore, the paw-withdrawal latencies in female rats at 24 h (5.9 ± 0.5 s) and 31 h (6.8 ± 0.6 s) after injection were also lower than those in male rats (24 h: 8.2 ± 0.4 s; 31 h: 9.6 ± 0.3 s). This result suggested that recovery from hyperalgesia was slower in female rats than in male rats. The overall hyperalgesia recovery curve obtained for female rats was lower than that obtained for male rats, suggesting that hyperalgesia is potentially more severe in female rats than in male rats.

### Sex difference in carrageenan-induced paw oedema

**Fig 2:**
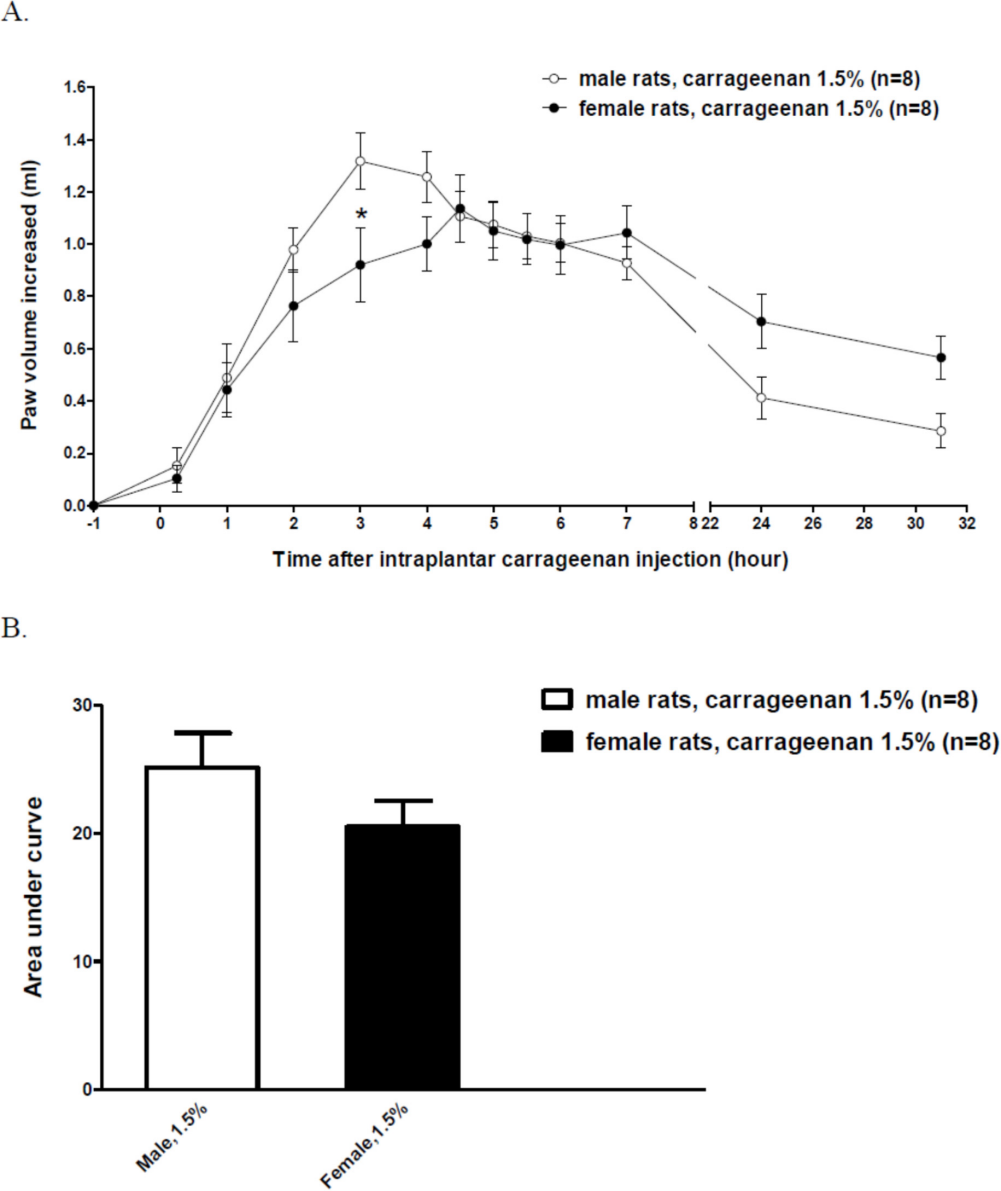
Comparison of paw edema induced by intraplantar carrageenan (1.5%) injections in male and female rats. Data from male and female rats received i.t. injections of saline were placed together for comparison. (A) The curves of carrageenan-induced increases of paw volume over time. Two-way ANOVA followed by Bonferroni post-test was used to analyze the statistical significance (*P < 0.05, female *vs*. male group). (B) Area under curve (AUC) obtained from the curves as shown in (A). AUC was only calculated for 8 hours (from -1 to 7 hr).

Female rats exhibited a slightly weaker paw oedema than male rats did when the animals received 1.5%-carrageenan injections (Fig 2A). The data revealed that at 3 h after intraplantar carrageenan-injection, the volume of the injected paw was lesser in female rats than in male rats (0.9 ± 0.1 ml *vs*. 1.3 ± 0.1 ml). At all the other time points, no statistically significant differences were detected between the male and female groups. However, compared with male rats, the female rats appeared to exhibit milder paw oedema at an early phase (2–4 h) after injection, whereas at a late phase (7–31 h), the females exhibited slower recovery from paw oedema. At 4.5–6 h, the period at which paw oedema was most severe in female rats, the increase in their paw volume was almost the same as the increase measured in male rats. The maximal increase in paw volume after injection was at 3 h in male rats, but at 4.5 h in female rats. To further quantify the severity of paw oedema to perform comparisons, we analyzed the area under curve (AUC) obtained from the curves shown in Fig 2A. The curves were only used from -1 to 7 h to prevent the over estimation at the later time points that occurred after considerably long intervals. However, our results revealed no statistically significant difference in the AUCs obtained for male rats (25.1 ± 2.7 ml × h) and female rats (20.5 ± 2.0 ml × h) (Fig 2B).

### Effect of Oxytocin (i.t.) on Hyperalgesia in Male and Female Rats

**Fig 3:**
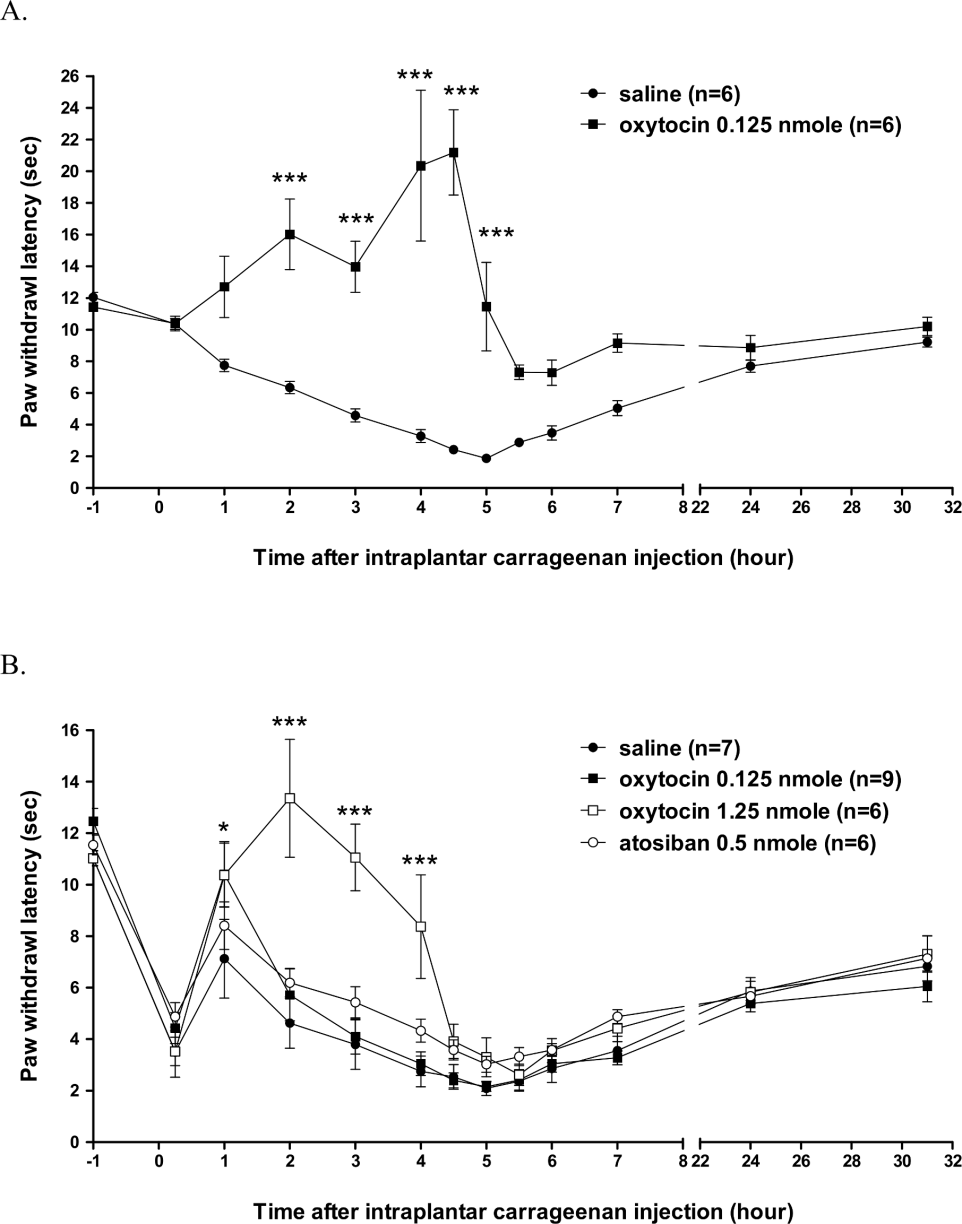
**Effects of intrathecally injected oxytocin on thermal hyperalgesia induced by intraplantar carrageenan injections in male (A) and female (B) rats.** The effect of atosiban in female rats was also shown in (B). The doses of i.t. injections in each group were as shown in the data sets on the top right corner of the figure. Two-way ANOVA followed by Bonferroni post-test was used to analyze the statistical significance (*P < 0.05, ***p < 0.001, oxytocin 0.125 nmole/1.25 nmole *vs*. saline group).

In male rats, intraplantar injection of 1.5% of carrageenan caused a significant hyperalgesia (Fig 3A). In the saline group, the paw withdrawal latency was gradually decreased over time following carrageenan injection. At the time point of 5 h after injection, the latency reached to the lowest value of 1.9 ± 0.2 s. Afterwards, the paw withdrawal latency gradually increased over time. At the time of 31 h after paw injection, the latency came back to 9.2 ± 0.3 s, which is close to the value at 0 h (10.4 ± 0.3 s). In the group received i.t. 0.125 nmol oxytocin injection, oxytocin caused a significant increase of paw withdrawal latency from 2 h to 5 h following carrageenan injection. At 4.5 h, the latency was increased to 21.2 ± 2.7 s as the maximal value, indicating a clear anti-hyperalgesia by oxytocin. These results were similar to those published in our previous report [22]

In the female rats that were injected i.t. with 0.125 nmol OT, a marked anti-hyperalgesic effect of OT was not observed, and no significant differences were detected when compared with the saline group at any time point except at 1 h. At 1 h after injection, the paw-withdrawal latency was longer in OT 0.125 nmol group than in the saline group (10.4 ± 1.3 s *vs*. 7.1 ± 1.5 s). However, OT clearly produced an anti-hyperalgesic effect when the dose increased to 1.25 nmol (OT 1.25 nmol group). The paw-withdrawal latencies were significantly longer than the basal values (11.0 ± 0.3 s) at 2, 3, and 4 h following carrageenan injection; at these time points, the latencies obtained after OT treatment and the latencies measured for the saline group were follows: 2 h: 13.4 ± 2.3 s *vs*. 4.6 ± 1.0 s; 3 h: 11.1 ± 1.3 s *vs*. 3.8 ± 1.0 s; 4 h: 8.4 ± 2.0 s *vs*. 2.8 ± 0.6 s. Beyond 4 h, the withdrawal latencies measured for the OT and saline groups were not significantly different. To examine whether endogenous OT exerted an effect, rats were injected (i.t.) with the OT-R antagonist atosiban (atosiban 0.5 nmol group); however, at this dose, atosiban did not markedly affect hyperalgesia in the rats (Fig 3). This result indicated that OT did not play a role or play a much minor role in hyperalgesia at the spinal level in female rats.

Intraplantar injection of 1.5% of carrageenan also potently induced hyperalgesia in the female rats in the control group, which received i.t. injections of saline (Fig 3B). The basal value of paw-withdrawal latency was between 11.0 and 12.5 s in four groups (saline, OT 0.125 nmol, OT 1.25 nmol, and atosiban 0.5 nmol). In the saline group, the basal value was 11.0 ± 0.3 s. After the carrageenan injection, the paw-withdrawal latency decreased at 15 min to 3.5 ± 1.0 s, but increased at 1 h to 7.1 ± 1.5 s, and then decreased again until reaching the minimal time of 2.1 ± 0.3 s at 5 h. From 5 to 7 h after injection, the latency gradually increased (5.5 h: 2.4 ± 0.4 s; 6 h: 2.9 ± 0.5 s; 7 h: 3.6 ± 0.6 s), and then increased further but did not return to the basal level even at 24 h (5.9 ± 0.5 s) and 31 h (6.8 ± 0.6 s) after the injection. This result indicated that the injection of 1.5% carrageenan induced severe hyperalgesia in female rats, much as it did in male rats.

### Anti-hyperalgesia induced by paraventricular nucleus stimulation in male and female rats

**Fig 4:**
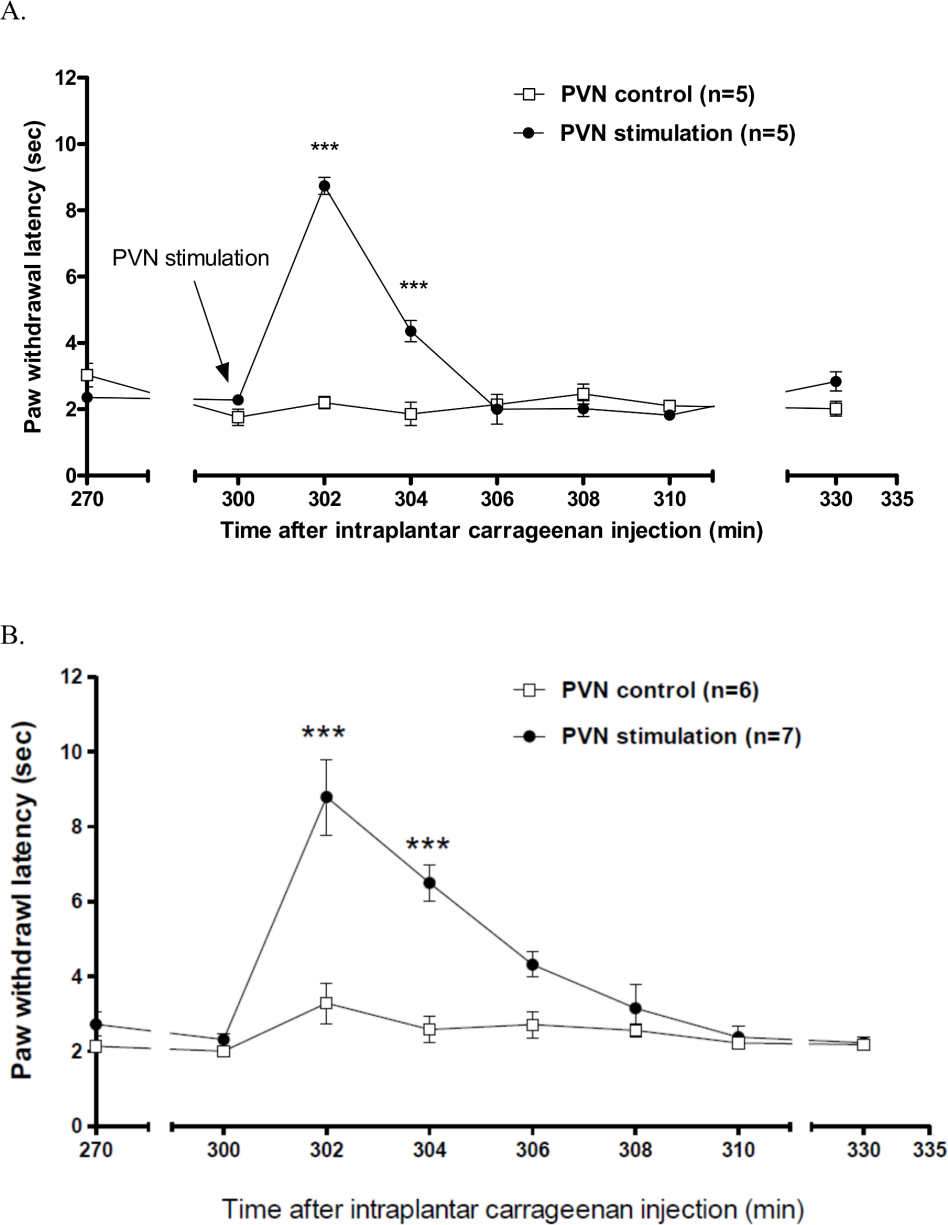
**Effects of PVN stimulation on thermal hyperalgesia induced by intraplantar carrageenan injections in male (A) and female (B) rats.** Two-way ANOVA followed by Bonferroni post-test was used to analyze the statistical significance (***P < 0.001, PVN stimulation *vs*. PVN control group).

In male rats (Fig 4A), PVN stimulation caused a clear but short anti-hyperalgesia effect. At the time point of 300 min (5 h) following intraplantar carrageenan injection, the PVN stimulations were given to the animals. Two minutes after PVN stimulation, the paw withdrawal latency was significantly increased to 8.7 ± 0.3 s, whereas the corresponding basal latency was 2.4 ± 0.1 s. At 4 minutes after PVN stimulation, the latency was still longer than its corresponding basal value (the latency before PVN stimulation) (4.4 ± 0.3 sec *vs*. 2.4 ± 0.1 sec). Six minutes after PVN stimulation, the latency returned to 2.0 ± 0.4 sec, which was similar to the basal value. This anti-hyperalgesic effect by PVN stimulation was similar to that we detected and reported previously [22].

Electrical stimulation in the PVN also strongly induced anti-hyperalgesia in female rats (Fig 4B). PVN stimulation was performed at the fifth hour (300 min) after carrageenan injections, and paw-withdrawal latencies were measured once every 2 min after PVN stimulation. The latencies increased from a basal value of 2.3 ± 0.2 s at 300 min to 8.8 ± 1.0 and 6.5 ± 0.5 s at 302 and 304 min, respectively. At 306 min, the latency decreased to 4.3 ± 0.3 s, but the difference between this and the latency in the control group (no PVN stimulation) was not statistically significant. The effects lasted for a short duration (4–6 min) and the efficacies of PVN stimulation were similar (male *vs*. female at 302 min: 8.7 ± 0.3 s *vs*. 8.8 ± 1.0 s)

### Quantitative difference in IRAP expression in the spinal cord of male and female rats

**Fig 5:**
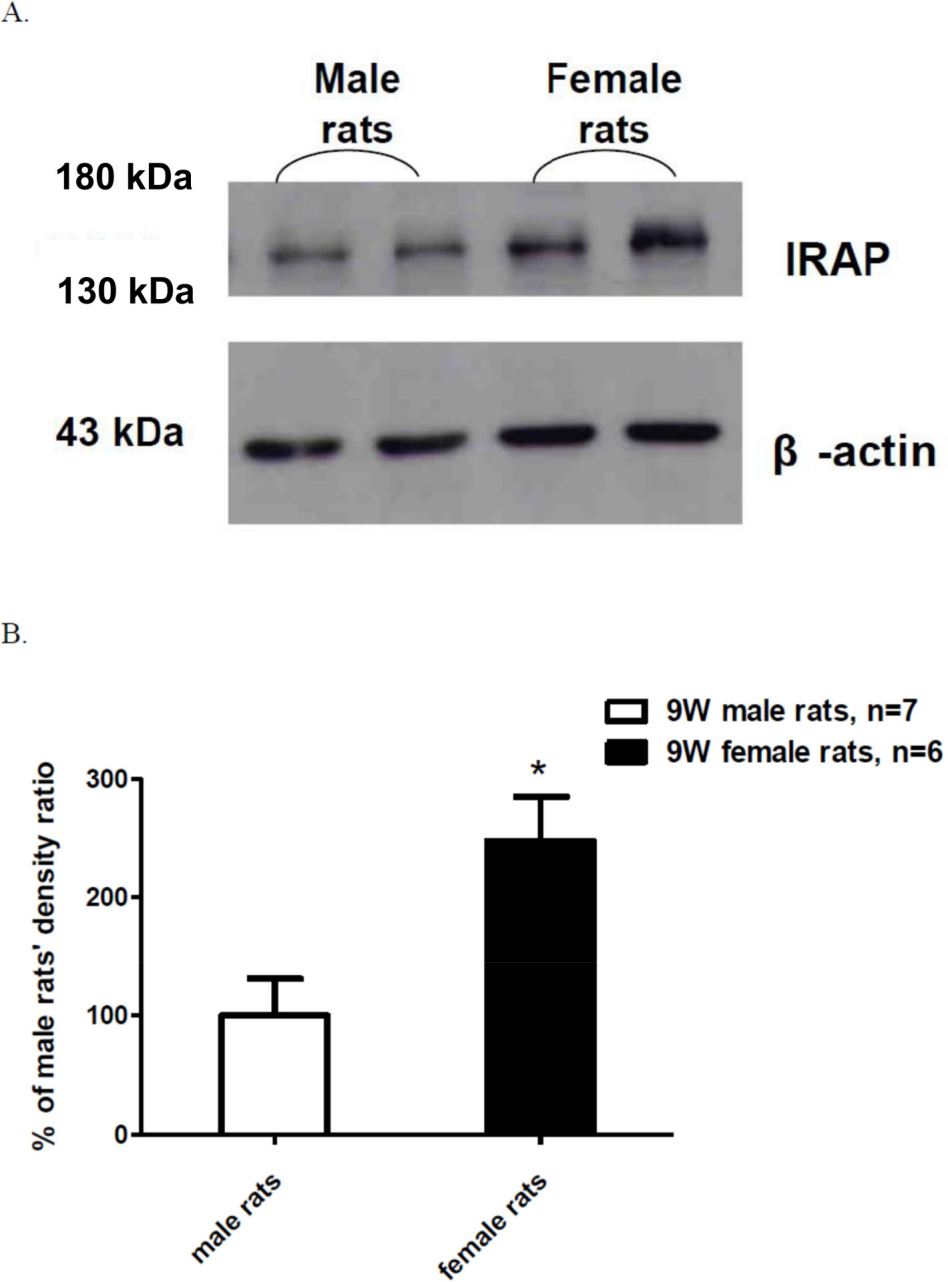
**(A) An examples of western blotting for IRAP expression in the lumbar spinal cord of male and female rats which were both nine weeks old. (B) The quantitative results of IRAP expression in the lumbar spinal cord of male and female rats.** Un-paired Student's *t*-test was used to analyze the statistical significance (*P < 0.05, female *vs*. male group).

In the lumbar spinal-cord (L1 to L6) tissue, the expression of IRAP was considerably higher in female rats than in male rats (Fig 5A). These data were obtained using spinal cords collected from 9-wk-old male and female rats that were littermates, which can rule out variations caused by the genetic background when comparing sex differences. Quantification of the western-blotting results (Fig 5B) showed that IRAP expression in the spinal cords of female rats was 247.5% ± 37.2%, as compared with 100% in the spinal cords of male rats. This result suggested that IRAP expression in the spinal cords of female rats was approximately 2.5-times higher than that in the spinal cords of male rats.

## Discussion

In this study, we first demonstrated a sex difference in OT-induced anti-hyperalgesia at the spinal level in female rats with intraplantar carrageenan-induced inflammation. Previously, we reported that i.t. injection of OT (0.125 nmol) strongly and effectively induced anti-hyperalgesia in male rats with carrageenan-induced inflammation [22]. By contrast, i.t. injection of OT did not strongly induce anti-hyperalgesia in female rats, except when OT was used at an extremely high dose. However, the female rats exhibited a temporary motor impairment lasting for 3 to 5 minutes following OT (1.25 nmol) injection. This may suggest a non-specific or even toxic effect of OT, although it is reversible. In male rats [22], the i.t. injections of OT were administered immediately after intraplantar carrageenan injections, the anti-hyperalgesia induced by OT lasted for over five hours. This result suggested that OT pre-treatment can prevent the occurrence of inflammatory hyperalgesia. Although the underlying mechanism of the OT effect remains unknown, the results indicated that OT can potentially be used for treating/preventing hyperalgesia, which can help to prevent post-operative pain. In translational studies, one report showed a clear anti-hyperalgesic effect of i.t. administration of OT in patients with low back pain [9]. Although OT exerted a strong therapeutic effect on both chronic and acute low back pain in this study, potential gender differences in OT-induced anti-hyperalgesia at the spinal level were not addressed. Our current results indicate that i.t. administration of OT might be less effective in treating hyperalgesia in female patients than in male patients. Although this suggests a limitation in the clinical use of OT, whether OT is ineffective in female patients with hyperalgesia should be tested in advance.

To investigate potential differences in the severity of carrageenan-induced hyperalgesia between male and female rats, we conducted paw oedema tests to measure the paw volumes of the animals before and after intraplantar injections. Our results showed that the overall severity of hyperalgesia was similar in male and female rats when the same dose of carrageenan was injected (1.5%, 100 μl). When compared with male rats, the female rats appeared to exhibit less severe paw oedema at early stages (before 4.5 h) of hyperalgesia and then recovered more slowly at late stages (after 7 h); however, the swelling of the carrageenan-injected paw was almost identical from 4.5 to 5.5 h after injection, the period during which hyperalgesia was most severe in both male and female rats. This can help eliminate the possibility that sex-dependent differences in the levels of peripheral inflammation affect OT-induced anti-hyperalgesia. Thus, the sex difference observed in OT-induced anti-hyperalgesia was not caused by peripheral inflammation being more severe in female rats than in male rats: the difference was likely caused by some other intrinsic feature of the spinal cord.

Paw-withdrawal latency in female rats decreased sharply at 15 min (0.25 h) after carrageenan injection. This phenomenon was not observed in male rats. Moreover, we noted that after carrageenan injections, male rats calmed down more quickly than female rats did. Therefore, the paw-withdrawal latency could be measured immediately after injections in male rats, but the female rats could be tested only after 15 min. This observation suggested that female rats are more sensitive to the acute pain caused by intraplantar carrageenan injections, which could have caused the decrease in paw-withdrawal latency at 0.25 h. However, the paw-withdrawal latency in female rats increased to around 7 s at 1 h, which is similar to the value obtained for male rats at 1 h; this result indicated that latency was revealed accurately in the absence of interference from the pain of injection. Examining the overall curves of hyperalgesia in male and female rats showed that the curve obtained for the female rats was slightly lower than that obtained for the male rats. However, these two curves showed no statistically significant difference. Thus, we speculate that the sex difference in OT-induced anti-hyperalgesia is not related entirely to the severity of hyperalgesia.

Our previous results suggested that the recently identified PVN-spinal cord OT neural pathway could be activated in male rats during the development of carrageenan-induced paw inflammation [22]; the pathway exhibits low activity under normal conditions. This finding is consistent with the results of Yu et al. [16]. Although PVN stimulation can also lead to the activation of magnocellular neurons and a peripheral release of oxytocin in the blood, the prompt onset and short duration of anti-hyperalgesia may indicate a direct activation on parvocellular neurons projecting to the spinal cord [19]. PVN stimulation also has been demonstrated to increase the release of oxytocin at the spinal cord in recent reports [34,35]. Therefore, we sought to investigate the physiological function of the PVN-spinal cord OT neural pathway in female rats. Our results showed that PVN stimulation induced a strong anti-hyperalgesic effect, and the difference in the effects of male and female rats was not statistically significant. This result indicated that the PVN-spinal cord OT neural pathway can be activated to produce anti-hyperalgesia in female rats by means of exogenous electrical stimulation. Thus, the absence of OT-induced anti-hyperalgesia in female rats could result from a low-level activation of the PVN-spinal cord OT neural pathway in response to peripheral inflammation. Exogenous stimulation at high intensity was sufficient for inducing anti-hyperalgesia in female rats.

In the search for the possible mechanism underlying the absence of OT-induced anti-hyperalgesia in female rats, the first clue was the low OT-R content in the spinal cord. In 2005, Uhl-Bronner et al. reported that OT-R expression in the spinal cord was higher in male rats than in female rats [27]; in this study, autoradiography was used to demonstrate that at all ages from postnatal day 0 to day 90, OT-R expression was higher in the spinal cords of male rats than of female rats. The difference was observed across spinal cord segments T13 to S4 and was quantified to be roughly 2-fold. This result could help explain why i.t. injection of OT did not induce anti-hyperalgesia in female rats. However, this cannot be the only explanation, because PVN stimulation also caused anti-hyperalgesia in female rats. Thus, we examined another factor, IRAP, which functions as an endogenous OT-degrading enzyme. Our results showed that the level of IRAP in the spinal cord was higher in female rats than in litter-matched male rats. This finding raises the possibility that OT is degraded more rapidly in the spinal cord of female rats than of male rats. This novel result offers another potential reason for the sex difference in OT-induced anti-hyperalgesia in rats. However, it is still unknown that whether IRAP is a rate limiting enzyme in the degradation of OT at the spinal cord. Apart from the tight regulation of IRAP on spinal OT, the possibility of different IRAP degradation, subcellular compartmentalization or glycosylation to cause sex difference should be also considered. In our preliminary data (not shown in the present study), we actually found a higher content of OT at the lumbar spinal cord of female rats when compared with those of male rats. This may indirectly support our hypothesis of higher IRAP expression and activity in female rats.

The results of this study indicate that a sex difference exists in OT-induced anti-hyperalgesia at the spinal level in rats; OT considerably less effective in female rats than in male rats. This finding suggests that i.t. injection of OT could be ineffective in preventing or treating hyperalgesia in female patients, although potential human-rat species differences in this effect remain to be elucidated. The sex difference likely arises from an intrinsic aspect of the OT system in pain regulation, and not because of distinct peripheral responses to inflammation in female and male rats. The lower OT-R expression and higher IRAP expression in the spinal cords of female rats than of male rats could both be responsible for the significant weaker effect of OT-induced anti-hyperalgesia in female rats. Our findings provide key insights that could facilitate future translational studies in which OT administration is used as a novel therapy for relieving pain.
